# Society Meetings

**Published:** 1894-11

**Authors:** 


### Society Meetings.

THE Medical Association of Central and Western New York held an interesting meeting at the Buffalo Academy of Medicine on Tuesday, October 16, 1894, under the presidency of Dr. Henry L.

Elsner, of Syracuse. The attendance was large and many of the papers were of a high order of merit. It is, however, a fair criticism to say that the time allowed for discussion was entirely inadequate, thus offering a poor incentive for authors to do good work. We hope this error will be rectified in future.

The business of such an association should all be done in committees, fewer papers should be read and longer discussions encouraged.

We begin in this number of the JOURNAL the publication of the proceedings of the association, which will be continued until all the papers are published. The president's address (page 204) is an able paper that deserves careful reading.

---

THE American Academy of Railway Surgeons will hold its first meeting in the parlors of the Grand Pacific Hotel, Chicago, Ill., on Friday and Saturday, November 9 and 10, 1894.

Dr. C. M. Daniels, of Buffalo, will read a paper on The Use of Horse Hair in Surgery, and Dr. C. B. Kibler, of Corry, Pa., will open the discussion on a paper by Dr. B. Merrill Ricketts, of Cincinnati, entitled The Environments of Railway Surgery.

---

### A COURSE IN BACTERIOLOGY.

THE faculty of the University of Buffalo desire to announce a Course in Bacteriology to be given in the Bacteriological Laboratory of the University during the six weeks beginning November 15, 1894. The course will comprise a study of the pathogenic and non-pathogenic bacteria and molds, in stained and unstained preparations and in plate cultures, with special reference in each instance to general microscopic and special bacteriological technique, including the various staining methods. The tubercle, typhoid, anthrax and tetanus bacilli and the cholera group of organisms will be studied in detail, and inoculation experiments made upon animals. Instruction will be given in the examination of air, earth, water and milk. Disinfection and sterilization will be elaborated and the method of testing the value of antiseptics studied. The present status of immunity will be briefly considered. The system employed in the Koch course in the hygienic institute in Berlin and that adopted by Professor Arnold in his laboratory at Heidelburg will be followed.

The course is open to second and third year students of the University and to all practitioners. Two hours will be devoted to work daily, and the time will be from 2 to 4 o'clock, or from 8 to 10 o'clock P. M., as determined by a majority of those entering. Those desiring to register will make application at once to Dr. Roswell Park, Director of Laboratory, or to Dr. Frank J. Thornbury, Demonstrator.

